# Supplementary material for: Transcriptome and Metabolome Analysis Reveal the Flavonoid Biosynthesis Mechanism of Abelmoschus manihot L. at Different Anthesis Stages
Source: Metabolites. 2023 Feb 1;13(2):216. doi: 10.3390/metabo13020216 (PMC9960708; doi:10.3390/metabo13020216)
Supplement: Supplementary file 1 [file metabolites-13-00216-s001.zip › metabolites-2035293-supplementary.pdf]

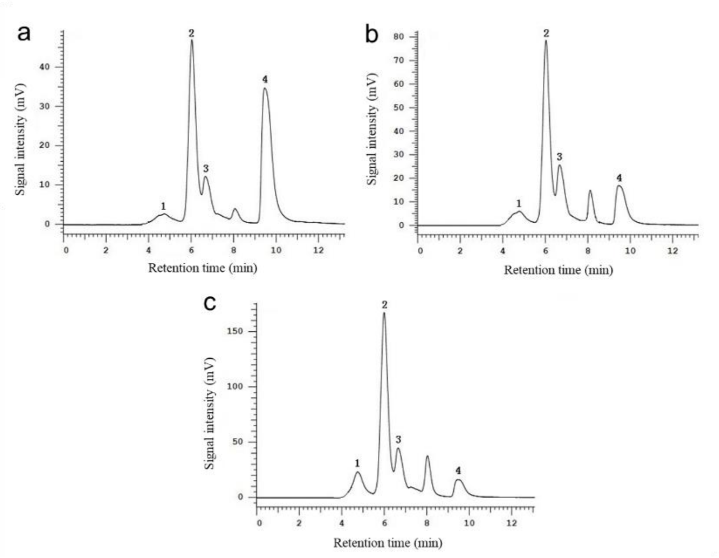

Figure S1. Chromatograms of flavonoids in *Abelmoschus manihot* L. at different flowering periods. a. T1 period, b. T2 period, c.T3 period. The peak numbers in the figure are represent vitexin rhamnoside (1), hyperoside (2), rutin (3), and quercetin (4).

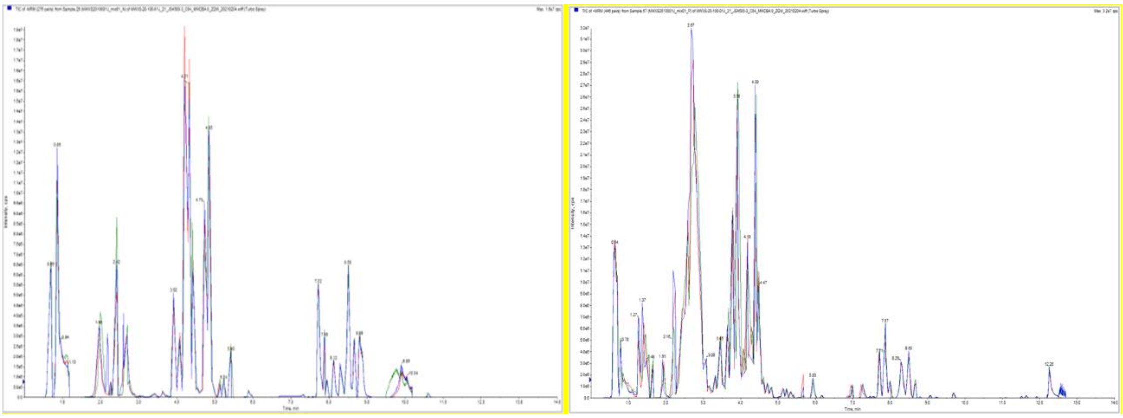

Figure S2. Overlap of TIC maps by mass spectrometry for QC samples.

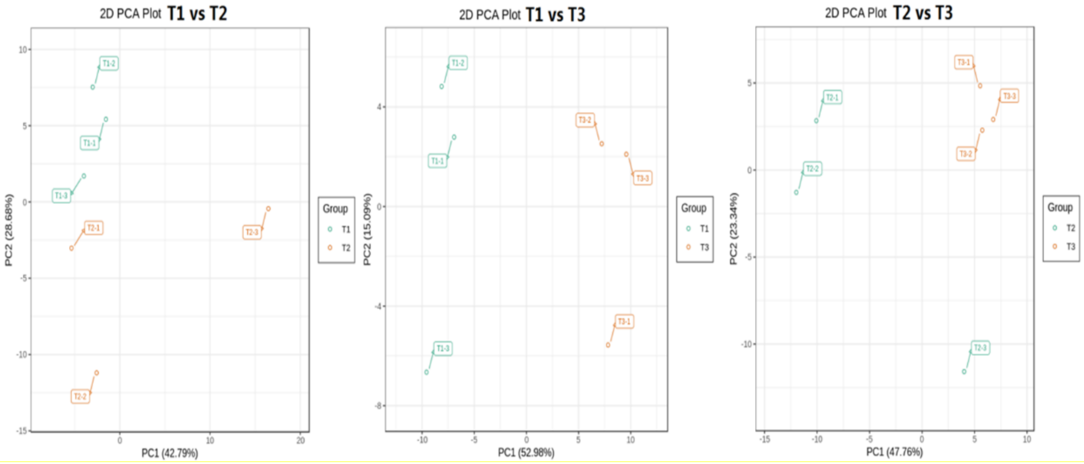

Figure S3. Grouped principal component analysis diagram of HSK flower groups.

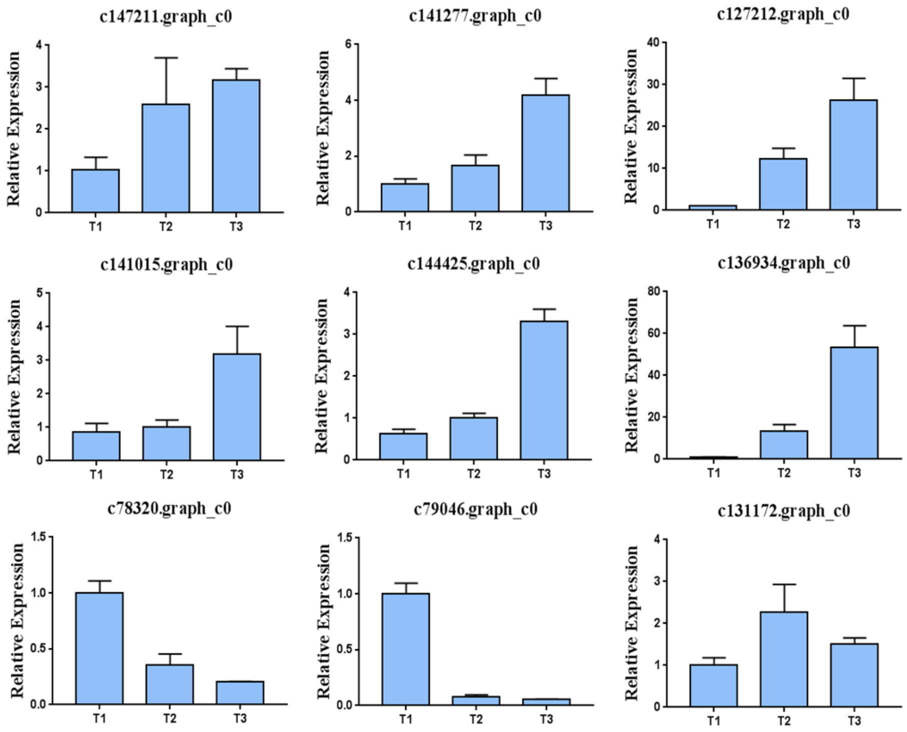

Figure S4. Expression analysis of 9 differentially expressed genes related to flavonoid and anthocyanin biosynthesis of *Abelmoschus manihot* L. by RT-PCR.

Table S1. Primer information in *Abelmoschus manihot* L.

Number	Gene name		Sequence（5'to3'）
1	c147211.graph_c0	Forward	AGCAGCCGAGTAAAAGTGTAGTG
		Reverse	GTTCCGCCTGTTCCTTGATAG
2	c141277.graph_c0	Forward	AGCAGCCGAGTAAAAGTGTAGTG
		Reverse	GTTCCGCCTGTTCCTTGATAG
3	c127212.graph_c0	Forward	CGACGGCACCAAAGGAAGA
		Reverse	GCGTTGAGGTATGACGGGAG
4	c141015.graph_c0	Forward	CCAGAAGTCCGACGAAGGTAG
		Reverse	CATCAGTTCATCTGTTTGCTTACC
5	c144425.graph_c0	Forward	GAGCAAAGAGTAGACAACGCAAGT
		Reverse	TCCGATGGCTACGAAGACG
6	c136934.graph_c0	Forward	CGAAAGTCTGGGATGAACCG
		Reverse	CCTGCCCAAACCAAAAGG
7	c78320.graph_c0	Forward	TTGGAGTGTACCTTGAACCTGAA
		Reverse	TCCGTATTGGGAACCTTTGAT
8	c79046.graph_c0	Forward	CAGATGTTTGTCGTTGGGCTTAG
		Reverse	GGCACGGCGGATAGTAGTTG
9	c131172.graph_c0	Forward	ATACACTGCTGGGGCTTGC
		Reverse	AGGGTTTCATCTTCTCGTCCA
10	AeActin	Forward	TCTTTCATCGGGATGGAAGC
		Reverse	ACTGAGCACAATGTTACCGTAGAG

Table S2. Retention time of flavonoids under chromatographic conditions.

Flavonoid components	Retention time（min）						RSD%
Vitexin rhamnoside	4.767	4.720	4.720	4.713	4.713	4.700	0.49
hyperoside	6.060	6.007	6.007	6.000	6.020	6.013	0.36
rutin	6.693	6.597	6.593	6.667	6.667	6.600	0.66
quercetin	9.367	9.333	9.333	9.327	9.340	9.327	0.16

Table S3. Statistics of clean data.

Sample	Number	Clean Data	Base Number	GC Content/%	%≥Q30/%	Mapped Reads	Mapped Ratio
	TI-1	29,954,755	8,975,679,212	44.63%	93.29%	22,652,772	75.62%
T1	TI-2	31,494,074	9,435,692,670	44.63%	93.15%	24,065,676	76.41%
	TI-3	25,895,161	7,757,881,574	45.05%	93.32%	19,952,827	77.05%
	T2-1	20,508,922	6,133,731,396	44.89%	92.79%	15,589,259	76.01%
T2	T2-2	27,340,890	8,188,656,882	44.64%	92.99%	21,121,627	77.25%
	T2-3	21,049,137	6,306,521,812	44.84%	93.01%	15,889,133	75.49%
	T3-1	27,398,536	8,209,257,194	46.33%	92.63%	19,634,228	71.66%
T3	T3-2	25,118,324	7,522,635,622	44.24%	92.89%	18,789,291	74.80%
	T3-3	23,132,799	6,928,801,232	45.38%	92.65%	16,841,642	72.80%
